# Supplementary material for: The potential of urban irrigation for counteracting carbon-climate feedback
Source: Nat Commun. 2024 Mar 18;15:2437. doi: 10.1038/s41467-024-46826-3 (PMC10948818; doi:10.1038/s41467-024-46826-3)
Supplement: Supplementary file 1 — Supplementary Information [file 41467_2024_46826_MOESM1_ESM.pdf]

Supplementary Information

for

The Potential of Urban Irrigation for Counteracting Carbon-Climate Feedback

Peiyuan Li[1,2], Zhi-Hua Wang[1], Chenghao Wang[3,4]

[1]*School of Sustainable Engineering and the Built Environment, Arizona State University, Tempe, USA*

[2]*Discovery Partners Institute, University of Illinois System, Chicago, USA*

[3]*School of Meteorology, University of Oklahoma, Norman, USA*

[4]*Department of Geography and Environmental Sustainability, University of Oklahoma, Norman, USA*

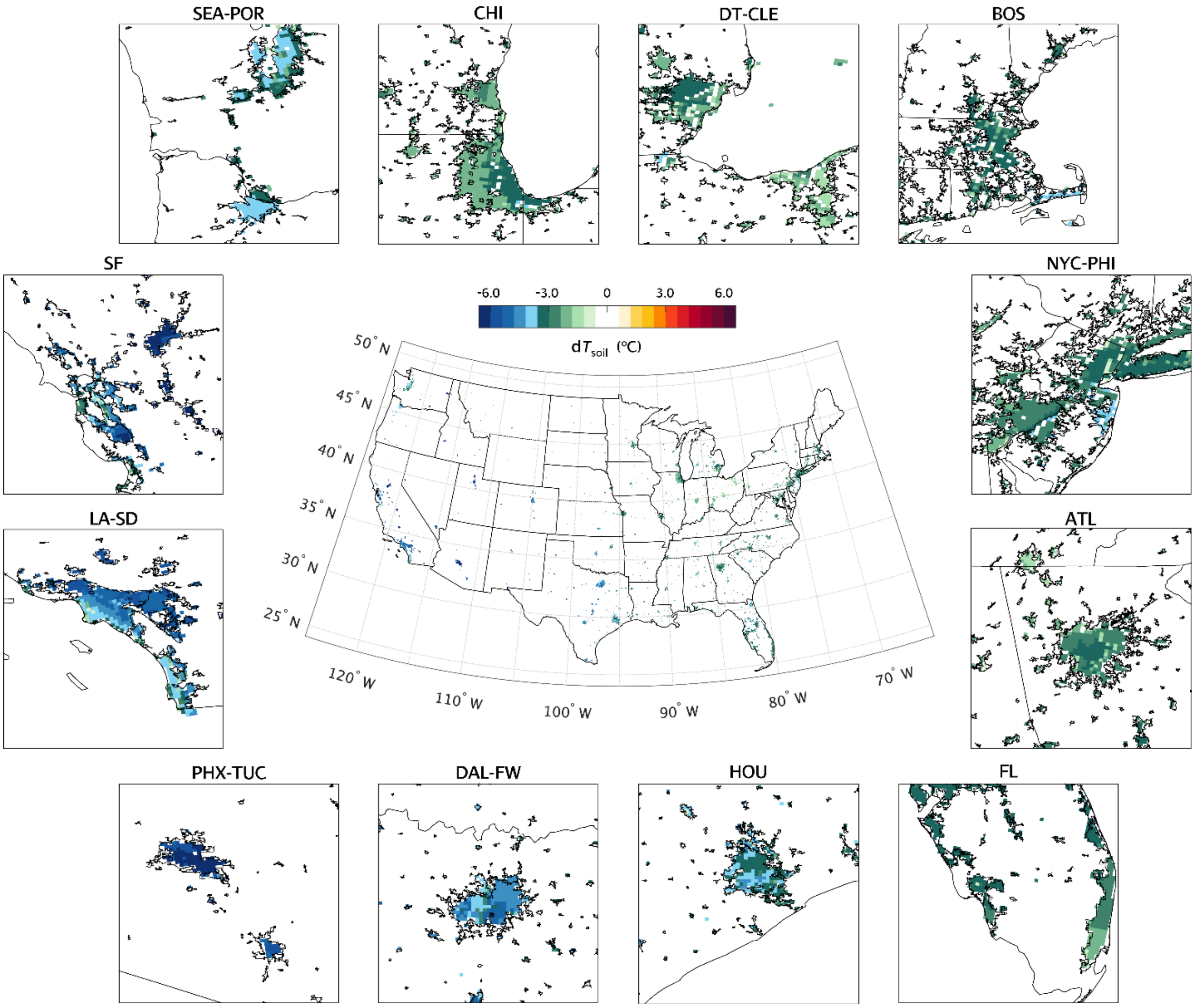

Supplementary Figure 1. Change of simulated topsoil temperature (dT_{soil}) after applying urban irrigation. Subplots around the CONUS map show the temperature change details over 12 metropolitan areas. SEA-POR: Seattle, WA and Portland, OR; CHI: Chicago, IL; DT-CLE: Detroit, MI and Cleveland, OH; BOS: Boston, MA; NYC-PHI: New York, NY and Philadelphia, PA; ATL: Atlanta, GA; FL: Cities around the coast of Florida; HOU: Houston, TX; DAL-FW: Dallas and Fort Worth, TX; PHX-TUC, Phoenix and Tucson, AZ; LA-SD: Los Angeles and San Diego, CA; SF: San Francisco, CA.

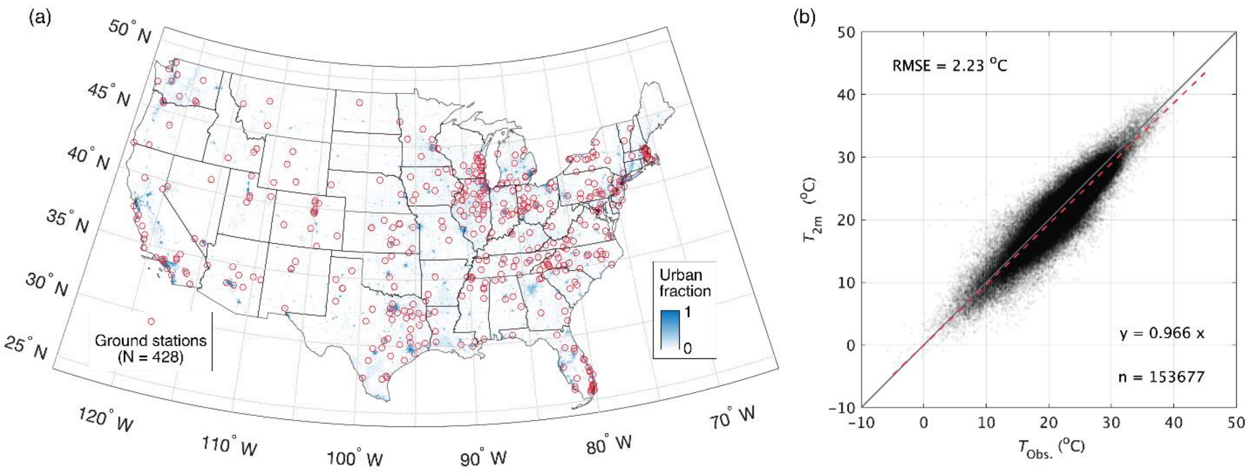

Supplementary Figure 2. (a) Urban fraction over CONUS and GHCNd ground stations used in this study for temperature validation; (b) comparison between WRF simulated daily mean 2-meter temperature (T_{2m}) and daily mean air temperature from GHCNd stations ($T_{obs.}$). Dashed red line is the ordinary linear regression of the data points (No. of stations, N= 428, No. of data points, n = 153677).

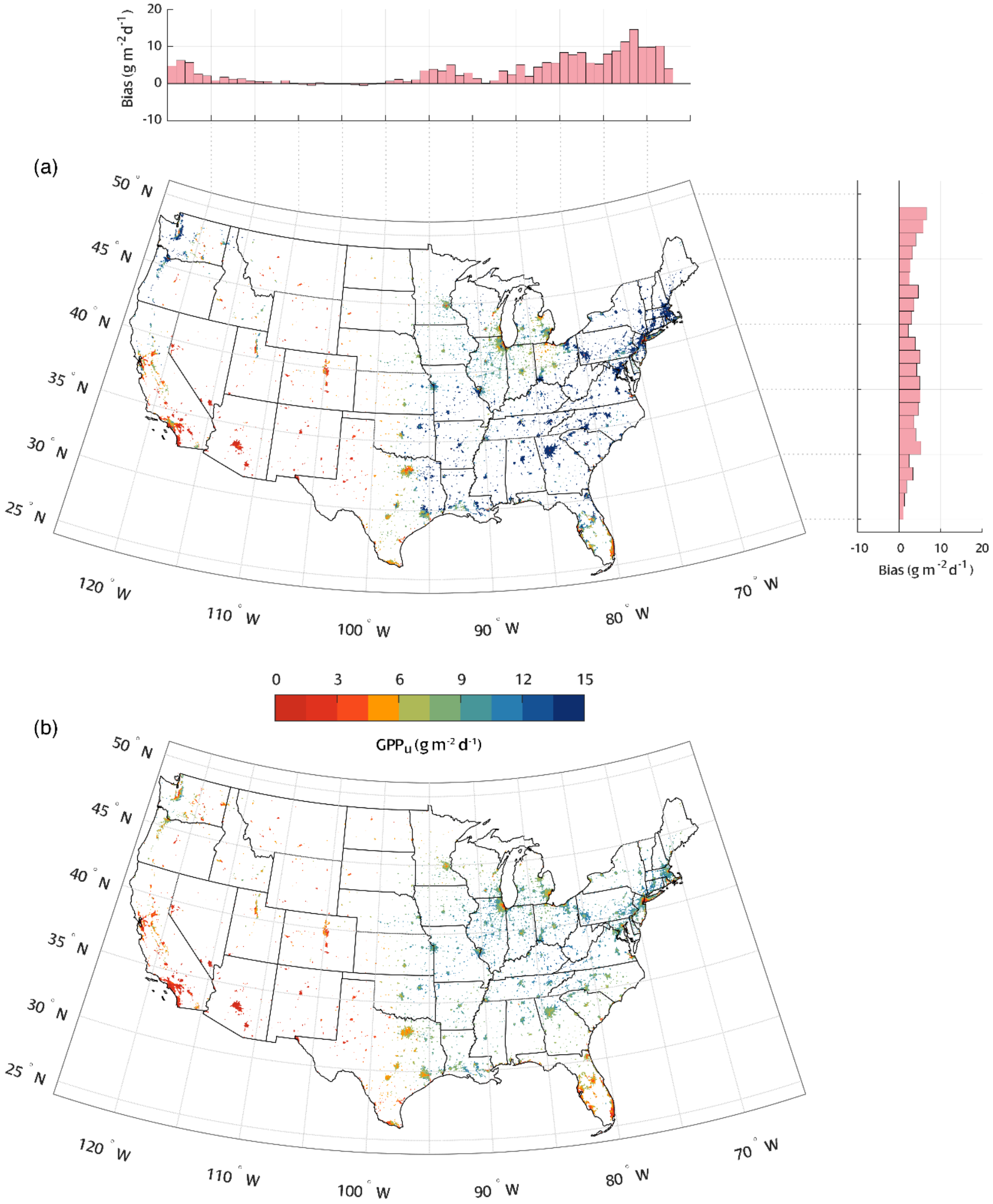

Supplementary Figure 3. Daily mean gross primary productivity (GPP) during summer months (May to August) in 2013 to 2015 from (a) model simulation in this study, and (b) VPM spatial gridded data. Histograms above and right to (a) show the difference between (a) and (b) in terms of longitude and latitude, respectively.

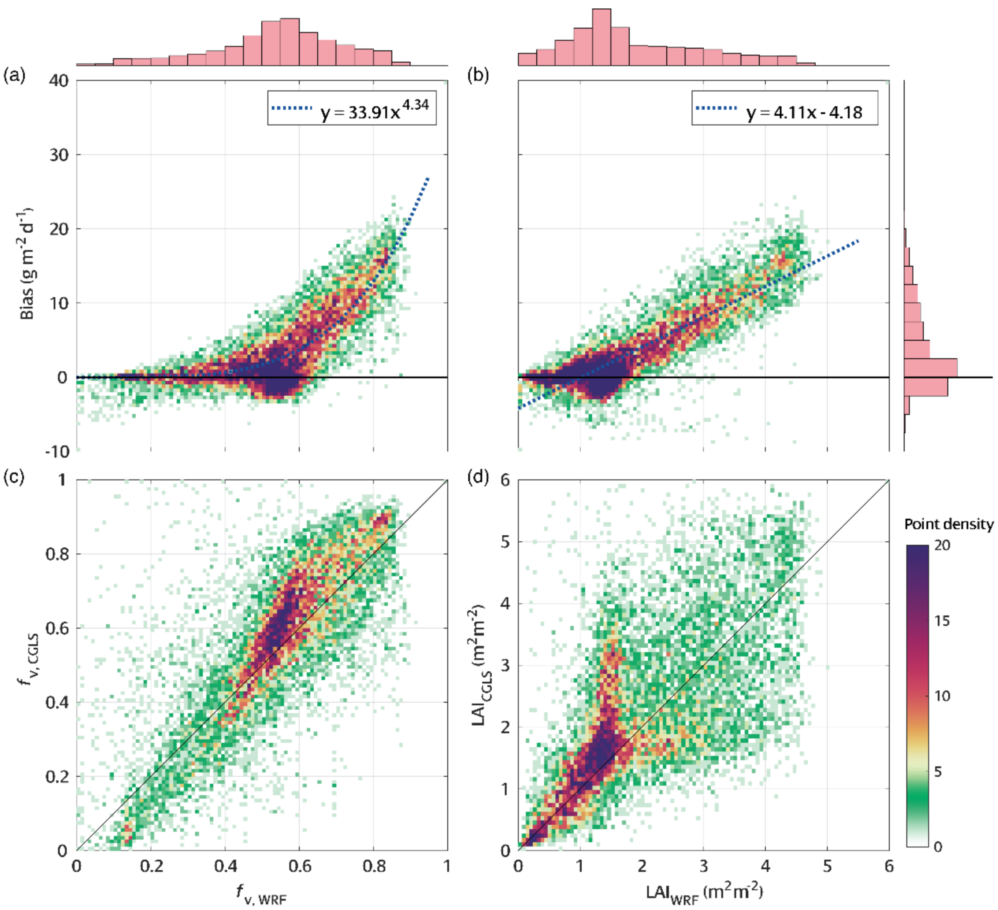

Supplementary Figure 4. Relations between (a) model bias and vegetation coverage (f_v); (b) model bias and leaf area index (LAI) in WRF. Comparison between values in WRF and CGLS of (c) vegetation coverage, and (d) leaf area index. Histograms over (a) and (b) show the distribution of f_v and LAI used in WRF. Histogram right to (b) shows the distribution of model bias. Filled color indicates the data point density in each subplot.

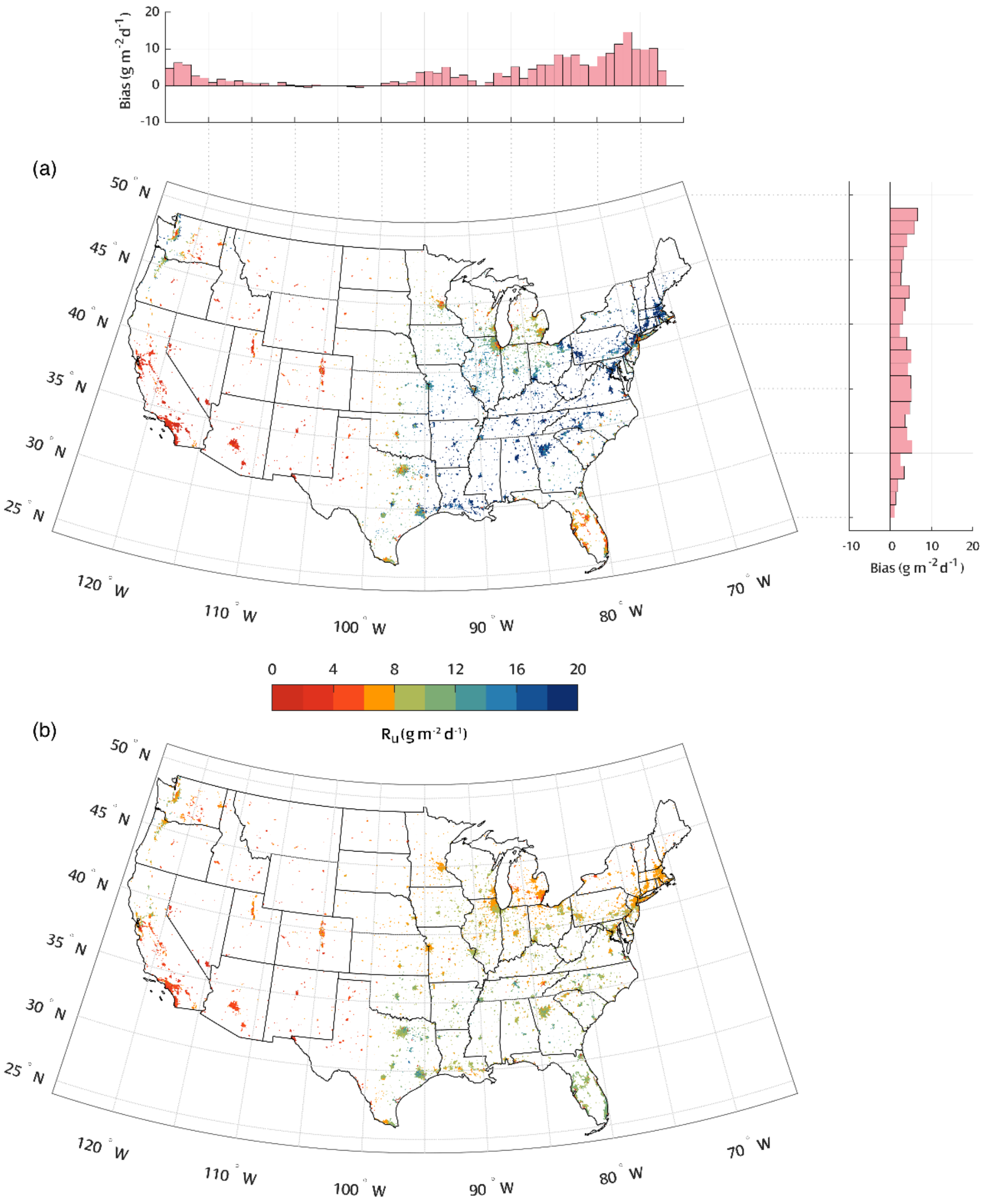

Supplementary Figure 5. Daily mean soil respiration during summer months (May to August) in 2013 to 2015 from (a) model simulation in this study; and (b) Soil Respiration Database (SRDB). Subplots above and right to (a) show the mean difference between (a) and (b) in terms of longitude and latitude, respectively.
